# Supplementary material for: Transcriptional linkage analysis with in vivo AAV-Perturb-seq
Source: Nature. 2023 Sep 20;622(7982):367–75. doi: 10.1038/s41586-023-06570-y (PMC10567566; doi:10.1038/s41586-023-06570-y)
Supplement: Supplementary file 1 — Reporting Summary [file 41586_2023_6570_MOESM1_ESM.pdf]

nature portfolio

Corresponding author(s): Randall J. Platt

Last updated by author(s): Jul 7, 2023

Reporting Summary

Nature Portfolio wishes to improve the reproducibility of the work that we publish. This form provides structure for consistency and transparency in reporting. For further information on Nature Portfolio policies, see our Editorial Policies and the Editorial Policy Checklist.

Statistics

For all statistical analyses, confirm that the following items are present in the figure legend, table legend, main text, or Methods section.

n/a	Confirmed	
☐	☒	The exact sample size (*n*) for each experimental group/condition, given as a discrete number and unit of measurement
☐	☒	A statement on whether measurements were taken from distinct samples or whether the same sample was measured repeatedly
☐	☒	The statistical test(s) used AND whether they are one- or two-sided<br>*Only common tests should be described solely by name; describe more complex techniques in the Methods section.*
☐	☒	A description of all covariates tested
☐	☒	A description of any assumptions or corrections, such as tests of normality and adjustment for multiple comparisons
☐	☒	A full description of the statistical parameters including central tendency (e.g. means) or other basic estimates (e.g. regression coefficient) AND variation (e.g. standard deviation) or associated estimates of uncertainty (e.g. confidence intervals)
☐	☒	For null hypothesis testing, the test statistic (e.g. *F*, *t*, *r*) with confidence intervals, effect sizes, degrees of freedom and *P* value noted<br>*Give P values as exact values whenever suitable.*
☒	☐	For Bayesian analysis, information on the choice of priors and Markov chain Monte Carlo settings
☒	☐	For hierarchical and complex designs, identification of the appropriate level for tests and full reporting of outcomes
☐	☒	Estimates of effect sizes (e.g. Cohen's *d*, Pearson's *r*), indicating how they were calculated

Our web collection on statistics for biologists contains articles on many of the points above.

Software and code

Policy information about availability of computer code

Data collection	Data were collected using publicly available software, as referenced in the methods section. RNA and gRNA UMI count matrices were generated from raw sequencing data using CellRanger v5.0 (10x Genomics).
Data analysis	FANS data were analyzed with FlowJo v10.5.0. Analyses were done using publicly available R packages (BOWTIE 2 v2.3.5, Seurat v3.0, EdgeR v3.36.0, MASS v7.3-50, UWOT v0.1.8, Augur v1.0.0, g:Profiler v0.2.0, destiny v3.17, CRISPResso2 v2.0.20, Enrichr v2.1, fgsea v3.17). Custom made scripts are available through the Platt Lab GitHub (https://github.com/plattlab/AAV-Perturb-seq). See methods section for detailed information.

For manuscripts utilizing custom algorithms or software that are central to the research but not yet described in published literature, software must be made available to editors and reviewers. We strongly encourage code deposition in a community repository (e.g. GitHub). See the Nature Portfolio guidelines for submitting code & software for further information.

Data

Policy information about availability of data

All manuscripts must include a data availability statement. This statement should provide the following information, where applicable:

- Accession codes, unique identifiers, or web links for publicly available datasets
- A description of any restrictions on data availability
- For clinical datasets or third party data, please ensure that the statement adheres to our policy

Raw and processed sequencing data generated for this study are available through the Gene Expression Omnibus (GEO accession number GSE236519).

Human research participants

Policy information about studies involving human research participants and Sex and Gender in Research.

Reporting on sex and gender	N/A
Population characteristics	N/A
Recruitment	N/A
Ethics oversight	N/A

Note that full information on the approval of the study protocol must also be provided in the manuscript.

Field-specific reporting

Please select the one below that is the best fit for your research. If you are not sure, read the appropriate sections before making your selection.

☒ Life sciences ☐ Behavioural & social sciences ☐ Ecological, evolutionary & environmental sciences

For a reference copy of the document with all sections, see nature.com/documents/nr-reporting-summary-flat.pdf

Life sciences study design

All studies must disclose on these points even when the disclosure is negative.

Sample size	For the pooled screen, the brains of 15 LSL-Cas9 mice injected with a AAV library targeting 22q11.2 genes. This number of animals permits sufficient infected nuclei after sorting for GFP+ nuclei. To analyze the 22q11.2 animal model, 30,000 nuclei from 3 WT and 3 LgDel mice were sequence.
Data exclusions	Cells with low number of RNA counts (< 1000) or gene counts (< 500) were removed. Cells with 0 or more than 1 detected gRNA were removed.
Replication	Representation of gRNA molecules inside the gRNA library was confirmed by deep sequencing. Cas9 activity was confirmed by indel analysis. The truthfulness of our AAV-Perturb-seq experiments was confirmed by arrayed injections. Once the method was implemented, all attempts at replication were successful.
Randomization	LSL-Cas9 animals were randomly injected and kept in populations of 2 to 4 animals per cage. LgDel animals were genotype to confirm zygosity and alocated to experimental group WT (LgDel+/+) or LgDel (+/-) depending on the genotype.
Blinding	LSL-Cas9 mice selection was done in a blinded fashion. Animals were selected by the support team at the animal facility. Immunohistochemistry and the tri-color experiment data analysos were done in a blinded fashion. Single cell data analyses were not performed in a blinded fashion to support the development of new analysis methods.

Reporting for specific materials, systems and methods

We require information from authors about some types of materials, experimental systems and methods used in many studies. Here, indicate whether each material, system or method listed is relevant to your study. If you are not sure if a list item applies to your research, read the appropriate section before selecting a response.

Materials & experimental systems

n/a	Involved in the study
☒	☐ Antibodies
☐	☒ Eukaryotic cell lines
☒	☐ Palaeontology and archaeology
☐	☒ Animals and other organisms
☒	☐ Clinical data
☒	☐ Dual use research of concern

Methods

n/a	Involved in the study
☒	☐ ChIP-seq
☐	☒ Flow cytometry
☒	☐ MRI-based neuroimaging

Eukaryotic cell lines

Policy information about cell lines and Sex and Gender in Research

Cell line source(s)	HEK293T cells were acquired from Sigma-Aldrich.
Authentication	HEK293T cells have been authenticated by the original vendors using short tandem repeat analysis.
Mycoplasma contamination	HEK293T cells were checked for mycoplasma every 3 months and tested negative throughout the study.
Commonly misidentified lines (See ICLAC register)	No misidentified cell lines were used in this study.

Animals and other research organisms

Policy information about studies involving animals; ARRIVE guidelines recommended for reporting animal research, and Sex and Gender in Research

Laboratory animals	Male LSL-Cas9, dCas9-KRAB, and LgDel mouse models between 6 and 8 weeks of age. Mice were kept under specific pathogen-free conditions on a standard light cycle, temperature, and humidity environment.
Wild animals	No wild animals were used in this study.
Reporting on sex	We used male mice as brain disorders such as ASD and Schizophrenia tend to have an higher prevalence in males.
Field-collected samples	No field-collected samples were used in this study.
Ethics oversight	ETH Animal Welfare Office; University Basel Veterinary Office; Basel-Stadt Cantonal Veterinary Office (Switzerland)

Note that full information on the approval of the study protocol must also be provided in the manuscript.

Flow Cytometry

Plots

Confirm that:

☒ The axis labels state the marker and fluorochrome used (e.g. CD4-FITC).

☒ The axis scales are clearly visible. Include numbers along axes only for bottom left plot of group (a 'group' is an analysis of identical markers).

☒ All plots are contour plots with outliers or pseudocolor plots.

☒ A numerical value for number of cells or percentage (with statistics) is provided.

Methodology

Sample preparation	Nuclei isolated from mouse brain tissue was processed as described in the methods section
Instrument	SONY MA 900
Software	FlowJo
Cell population abundance	We sorted a minimum of 50,000 infected nuclei per condition.
Gating strategy	Nuclei was first gated with a DNA dye (Ruby dye, ThermoFisher). Single-nuclei were sorted based on GFP expression.

☒ Tick this box to confirm that a figure exemplifying the gating strategy is provided in the Supplementary Information.
